# Cryotherapy-Driven Modulation of Postoperative Pain in Single-Visit Endodontic Treatment Across Different Obturation Materials: A Retrospective Study

**DOI:** 10.3390/jcm15103899

**Published:** 2026-05-19

**Authors:** Kaan Ilıcalı, Ahter Şanal Çıkman, Özge Başar

**Affiliations:** 1 Lema Private Dental Clinic, 34290 Istanbul, Turkey; kaanilicali@gmail.com; 2 Department of Endodontics, Faculty of Dentistry, Recep Tayyip Erdogan University, 53100 Rize, Turkey; 3 Department of Endodontics, Faculty of Dentistry, Adnan Menderes University, 09100 Aydın, Turkey; basar.ozge@hotmail.com

**Keywords:** bioceramic, cryotherapy, epoxy resins, postoperative pain

## Abstract

**Background/Objectives**: This study aimed to evaluate the effect of intracanal cryotherapy on postoperative pain across obturation materials with different chemical compositions and physical properties in single-visit root canal treatment. **Methods**: Patients diagnosed with irreversible pulpitis (*n* = 73), treated in a single visit by the same operator, were categorized based on the obturation material used (AH Plus, TotalFill BC Sealer, and TotalFill BC RRM) and whether intracanal cryotherapy (20 mL of sterile saline at 4 °C for 5 min) was applied. Visual Analog Scale (VAS) scores obtained from patient follow-up forms at 24, 48, and 72 h were evaluated. **Results**: Cryotherapy (+) groups showed consistently lower pain scores at all time points compared with cryotherapy (−) groups (*p* < 0.001). Within the cryotherapy (+) groups, both TotalFill BC Sealer and TotalFill BC RRM exhibited significantly lower pain scores than AH Plus at 48 h (*p* < 0.05). In the cryotherapy (−) groups, TotalFill BC Sealer showed significantly lower pain scores on the third postoperative day (*p* < 0.05). **Conclusions**: Intracanal cryotherapy may serve as an effective adjunctive technique associated with lower early postoperative pain scores. Material-related differences became evident at 48 and 72 h, suggesting that obturation material selection may influence postoperative pain patterns and patient comfort during the later postoperative period.

## 1. Introduction

Postoperative pain is a common outcome of root canal treatment and is primarily associated with periapical inflammation; therefore, identifying its etiologic factors is essential for minimizing its occurrence [1]. Mechanical factors such as over-instrumentation, excessive apical preparation, and loss of apical constriction can promote the extrusion of debris and irritants into periapical tissues. Additionally, chemical agents, including irrigants or intracanal medicaments, may cause tissue irritation when they extend beyond the apical region [2,3]. Patient-related factors, including preoperative pain intensity, anxiety levels, and individual pain sensitivity, as well as microbial factors, may further influence the occurrence and severity of postoperative pain [4].

Various strategies aim to reduce postoperative pain by limiting periapical extrusion and improving patient comfort, including minimal apical enlargement, precise working length control, effective irrigation, and occlusal reduction [5,6]. In recent years, intracanal cryotherapy has been increasingly investigated as a non-pharmacological strategy to reduce pain and enhance patient comfort [7]. Its clinical applications have expanded across medical fields, including wound care, analgesia, sports injuries, and dermatological conditions [8,9]. More recently, its use has extended to dentistry for the management of postoperative symptoms [10]. In endodontics, intracanal cryotherapy—typically applied as cold saline during final irrigation—has been shown to reduce postoperative pain by inducing local vasoconstriction, slowing nerve conduction, and modulating inflammatory mediators, making it a simple and cost-effective adjunct to root canal treatment [11]. However, postoperative pain after root canal treatment is multifactorial and may also be influenced by other treatment-related variables including the instrumentation protocol, irrigation procedure, obturation technique, and the type and properties of the root canal filling material [12]. It has been noted that root canal instrumentation protocols, in particular, have a significant impact on postoperative pain [13]; therefore, standardization of instrumentation techniques is important to evaluate the effect of factors such as cryotherapy and filling material on postoperative pain.

Although intracanal cryotherapy has been shown to reduce postoperative pain, whether its analgesic effect is maintained across obturation materials with different chemical compositions and physical properties remains unclear. During the early setting phase of epoxy resin-based sealers, a small fraction of unreacted or partially reacted epoxy and amine components may be present at the material surface or released into surrounding tissues, where they can induce transient cytotoxic and pro-inflammatory responses [14]. This early biological interaction may contribute to nociceptor sensitization and postoperative discomfort [15]. Through a different mechanism, calcium silicate-based bioceramic sealers may contribute to postoperative discomfort via alkaline ion release during hydration and initial tissue reactions associated with biomineralization [16]. Beyond these biochemical differences, variations in viscosity and delivery form among bioceramic products (e.g., flowable sealers versus premixed putties) may influence the extent of material–tissue contact and the dynamics of potential apical extrusion, thereby diversifying the underlying pain mechanisms [17,18].

One of the most critical questions that remains unanswered in the current literature is whether the physiological suppression provided by intracanal cryotherapy offers a universal solution across these heterogeneous material–tissue-related pain pathways. Given the limited evidence addressing the combined effects of these interactions, this study aims to comparatively evaluate the impact of intracanal cryotherapy on the postoperative pain trajectory associated with obturation materials of different chemical compositions and viscosities, thereby defining the clinical boundaries of this adjunctive approach. This study tested two null hypotheses: (i) intracanal cryotherapy would not significantly reduce postoperative pain at 24, 48, and 72 h; and (ii) its effect would not vary among AH Plus, TotalFill BC Sealer, and TotalFill BC RRM or with differences in the viscosity and delivery form of bioceramic materials.

## 2. Materials and Methods

The study was conducted at the Department of Endodontics, Recep Tayyip Erdogan University (Ethics Committee Approval No: 2023/230), in accordance with the Declaration of Helsinki. All patients had previously provided written informed consent for treatment and for the use of their anonymized clinical and radiographic data for research purposes. This study was conducted according to Preferred Reporting items for Observational studies in Endodontics (PROBE) 2023 guidelines [19].

The main aims of this research were to examine the differences between six independent groups and the change within groups over time. A priori power analysis was performed using G*Power software (version 3.1.9.2). The expected standardized effect size was derived from a previous randomized clinical trial on intracanal cryotherapy and postoperative endodontic pain [20]. Based on this effect size (d = 1.6243), with α = 0.05 and 95% theoretical power, the minimum required sample size was calculated as 11 participants per group. This retrospective study included patients who underwent single-visit root canal treatment between September 2022 and September 2023 at the Department of Endodontics, Recep Tayyip Erdoğan University, using a predefined and standardized clinical protocol. Compliance was retrospectively verified using clinical records, radiographic documentation, and postoperative follow-up forms, and cases with incomplete or uncertain protocol adherence were excluded from the study.

A total of 513 patient follow-up forms (Supplementary Table S1) were screened, of which 122 patients diagnosed with irreversible pulpitis were identified. Eligible patients were aged 18 years or older, systemically healthy, and presented with severe preoperative pain (VAS > 60). Only single-rooted, single-canal vital teeth diagnosed with irreversible pulpitis and treated in a single visit were included. Patients were excluded if they had any systemic disease; had used analgesics within three days or antibiotics within seven days before or after treatment; or presented with advanced periodontal disease, intraoperative complications, radiographic evidence of inadequate obturation (overfilling or underfilling), preoperative percussion sensitivity, or if the treatment was not performed in accordance with the department’s standard clinical protocol (e.g., alternative instrumentation or activation systems, non-standard irrigation regimens, modified obturation techniques, or the administration of more than one local anesthetic injection). A final sample of 73 cases met the eligibility criteria and were included in the study. Patients were categorized according to both the type of obturation material applied; AH Plus (Dentsply Sirona, Charlotte, NC, USA), TotalFill BC Sealer (FKG Dentaire Sàrl, La Chaux-de-Fonds, Switzerland), and TotalFill BC RRM Paste (FKG Dentaire Sàrl, La Chaux-de-Fonds, Switzerland) and whether intracanal cryotherapy was performed. Selection of the obturation material and the application of intracanal cryotherapy reflected routine clinical decision-making and operator preference. Cases involving complex canal structures, resorptions, or complications did not meet the eligibility criteria of the study. Therefore, cases in which a specific obturation material or intracanal cryotherapy protocol had been selected due to case complexity, non-standard treatment protocols, or procedural complications were excluded from the analysis.

- AH Plus (*n* = 11).
- AH Plus + Cryotherapy (*n* = 11).
- TotalFill BC RRM (*n* = 11).
- TotalFill BC RRM + Cryotherapy (*n* = 13).
- TotalFill BC Sealer (*n* = 13).
- TotalFill BC Sealer + Cryotherapy (*n* = 14).

To ensure procedural standardization, only cases treated by the same operator (K.I.) according to the standard departmental protocol were included. All selected cases were managed under rubber-dam isolation following local anesthesia with 2 mL of articaine hydrochloride containing 1:100,000 epinephrine. Only cases in which the working length was determined using an electronic apex locator (Woodpex III; Woodpecker, Guilin, China) and confirmed by periapical radiography were included. Root canal shaping was performed using EndoArt Smart Gold rotary files (EndoArt, İstanbul, Turkey), and only teeth with a documented final apical preparation size of 40/0.04 were selected. The final irrigation sequence consisted of 17% ethylenediaminetetraacetic acid, followed by sterile saline and 2.5% sodium hypochlorite, with a final saline rinse. In cases where intracanal cryotherapy had been performed, the protocol consisted of final irrigation with 20 mL of sterile saline at 4 °C for 5 min using pre-chilled syringes to maintain the target temperature. The cold saline was delivered slowly in a continuous flow throughout the 5-min period using a 30-gauge side-vented irrigation needle positioned 2 mm short of the working length to maintain cooling efficacy while minimizing the risk of apical extrusion. Only cases obturated using the single-cone technique and restored with a light-cured composite resin (Palfique Estelite LX5, Tokuyama Dental Corporation, Tokyo Japan) were included in the study.

According to the routine clinical follow-up protocol, patients were provided with VAS forms after treatment and instructed to mark their pain levels at 24, 48, and 72 h. During the routine third-day clinical control, the treating clinician (K.I.) evaluated the marked forms and recorded the scores in patient follow-up forms. A change of ≥13 mm on the VAS was considered clinically relevant based on established pain research standards [21]. Data analysis was conducted in Jamovi (v2.3.28). Prior to statistical analyses, the normality of the data distribution was assessed using the Shapiro–Wilk test. Comparisons of VAS scores between cryotherapy (+) and cryotherapy (−) groups at each time point were performed using the Mann–Whitney U test. Differences among obturation materials within the same cryotherapy condition were analyzed using the Kruskal–Wallis test followed by the Dwass–Steel–Critchlow–Fligner post hoc procedure, and temporal changes in pain scores within each group were evaluated using the Friedman test.

## 3. Results

The study population included 51 females (69.9%) and 22 males (30.1%), with a mean age of 46.3 years (19–68 years). No statistically significant differences were observed among the groups in terms of age, sex, or preoperative pain scores (*p* > 0.05), indicating a homogeneous distribution across the groups. Postoperative pain scores were analyzed according to obturation material type and their comparison across time points. No statistically significant differences were observed among the evaluated obturation materials at any postoperative time point (*p* > 0.05). However, in all groups, postoperative pain decreased significantly over time compared with preoperative values (*p* < 0.001).

Table 1 shows that intracanal cryotherapy was associated with significantly lower postoperative pain scores compared with the non-cryotherapy group at all time points (Days 1–3; *p* < 0.001). Pain levels decreased significantly over time within both groups (*p* < 0.001).

**Figure 1:**
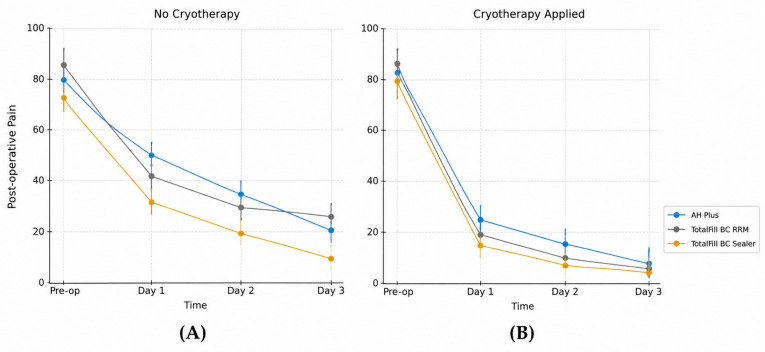
Temporal changes in postoperative pain (VAS scores) for different obturation materials: (**A**) no cryotherapy and (**B**) cryotherapy applied.

Table 2 demonstrates that intracanal cryotherapy was associated with significantly lower postoperative pain scores across all obturation materials at each time point (Days 1–3; *p* < 0.001), with consistent within-group decreases in pain over time for both cryotherapy and non-cryotherapy conditions (*p* < 0.001) (Figure 1).

While no significant differences were observed among materials under cryotherapy (+) conditions on Days 1 and 3, a material-dependent difference emerged on Day 2, with both TotalFill BC Sealer and TotalFill BC RRM exhibiting significantly lower pain scores than AH Plus (*p* < 0.05). In the cryotherapy (−) groups, a material-dependent difference was observed on Day 3, with the TotalFill BC Sealer group showing significantly lower pain scores than both AH Plus and TotalFill BC RRM (*p* < 0.05).

## 4. Discussion

Postoperative pain is a common outcome after endodontic procedures, primarily resulting from physical, chemical, and thermal irritation of periapical tissues throughout the treatment process—from access cavity preparation to root canal obturation [3]. Various strategies, including thorough debridement, multi-visit protocols, and pharmacological interventions (analgesics, NSAIDs, or steroids), have been proposed to manage pain; however, these drugs may be associated with gastrointestinal and cardiovascular adverse effects [22,23]. Intracanal cryotherapy is a simple, cost-effective, and clinically safe technique for managing postoperative pain after endodontic treatment. It reduces analgesic consumption, particularly during the first three postoperative days when pain typically peaks. Its analgesic effect is mediated by vasoconstriction and reduced nociceptive nerve activity, which limit inflammatory mediator release and slow neural pain transmission [24,25,26].

Retrospective analyses based on routinely collected clinical data are widely used in clinical research to generate reliable and clinically meaningful evidence for non-invasive adjunctive interventions. Although the retrospective design limits causal inference, this approach reflects real-world treatment conditions and enhances the external validity of the findings [27]. In the present study, only patient records of treatments performed by a single operator were included, and the use of systematically recorded clinical charts enabled a high level of standardization across cases, including uniform local anesthesia, instrumentation systems, irrigation protocols, final apical preparation size, and obturation techniques. In line with the approach adopted by Graunaite et al. [28] and Gondim et al. [29], only single-visit root canal treatments were included to minimize confounding factors such as intracanal medicament use and potential coronal leakage from temporary restorations, thereby enabling a more specific evaluation of the effects of cryotherapy and obturation materials on postoperative pain. Accordingly, given that postoperative pain typically peaks within the first 24–48 h and declines thereafter, pain assessments recorded in patient follow-up forms at Days 1, 2, and 3 were selected to capture the clinically relevant pain trajectory [30]. The significant reduction in postoperative pain observed over time within each obturation material group provides the primary context for interpreting these findings. This framing suggests that the observed decline in postoperative pain across all obturation materials reflects the combined influence of the natural healing process and the standardized clinical protocol rather than obturation material-specific effects [1]. The observed median VAS reductions of 10–20 mm fall within the range considered clinically meaningful for patient-perceived pain relief [21], underscoring the practical relevance of intracanal cryotherapy in routine single-visit endodontics.

In the present study, intracanal cryotherapy was associated with a reduction in postoperative pain at 24, 48, and 72 h across all obturation materials, indicating a largely material-independent pain-modulating effect. Accordingly, the first null hypothesis—that intracanal cryotherapy would not significantly reduce postoperative pain at these time points—was rejected. This finding suggests that cryotherapy may be considered as an adjunct in single-visit endodontic protocols, particularly in patients presenting with high baseline pain levels or heightened pain sensitivity, to enhance early postoperative comfort without altering the obturation strategy. These findings are consistent with those of Keskin et al., who reported significantly lower postoperative pain scores following single-visit root canal treatment in vital teeth treated with intracanal cryotherapy, particularly within the first 48 h [7]. Similarly, Vera et al. [11] demonstrated in a randomized multicenter clinical trial that the application of cold saline as a final irrigant significantly reduced both postoperative pain and analgesic intake in teeth with symptomatic apical periodontitis, supporting the clinical relevance of cryotherapy as an adjunctive pain-management strategy. In contrast, Bazaid and Kenawi [31] reported no significant analgesic benefit of cryotherapy in patients without apical periodontitis, which may be attributed to differences in the cryotherapy protocol, including the saline temperature used or shorter application durations, as well as the lack of homogeneity in baseline VAS scores across patient groups. In another randomized clinical trial, Ghabraei et al. [32] reported no sustained differences in postoperative pain between cryotherapy and control groups beyond the very early time point (6 h); this may be attributed to the use of postoperative analgesics in their cohort, which could have masked the measurable effect of intracanal cryotherapy. Among participants receiving cryotherapy, the lower Day-2 pain scores observed with bioceramic obturation materials may be associated with differences in material–tissue interaction rather than early procedural factors. Although bioceramic materials and AH Plus are applied in a flowable form and can form a thin sealer film, their biological behavior differs [33]. The calcium silicate-based composition of BC Sealer and RRM may promote a more favorable tissue response and faster resolution of local inflammation, whereas the epoxy resin-based chemistry of AH Plus may sustain a mild inflammatory stimulus during its setting phase [14]. Accordingly, although these material-related differences may be associated with lower nociceptor sensitization and reduced postoperative discomfort in the bioceramic groups at later time points, this interpretation should be considered only as a possible explanation for the observed clinical pain patterns rather than as a confirmed biological effect. This transient tissue response may help explain the higher pain scores observed for AH Plus on the second postoperative day under cryotherapy conditions, suggesting that cryotherapy attenuates but does not fully eliminate pain-modulating mechanisms associated with the obturation material. Accordingly, the second null hypothesis was rejected, as differences among AH Plus, TotalFill BC Sealer, and TotalFill BC RRM were detected under cryotherapy conditions on the second postoperative day. Unlike prior studies that primarily evaluated cryotherapy as a standalone intervention, the present study extends current evidence by suggesting a possible interaction with obturation material properties under standardized clinical conditions [7,11,31].

In the absence of cryotherapy on the third postoperative day, differences between the bioceramic materials became apparent. In an in vitro study evaluating TotalFill BC Sealer, TotalFill BC RRM, and an epoxy resin-based sealer under unset conditions, the authors reported that both material type and dilution significantly affected human osteoblast cell viability, mineralization potential, and cellular morphology [18]. Despite sharing a similar calcium silicate-based chemical composition [18], TotalFill BC Sealer and TotalFill BC RRM differ in their physical form, which may influence their interaction with periapical tissues [34]. BC Sealer is applied as a thin, flowable film along the canal walls, potentially resulting in more uniform adaptation and limited apical extrusion, whereas BC RRM was used in a paste form with higher viscosity, which may increase the likelihood of localized material accumulation near the apical region [17,35]. Such differences in viscosity and placement dynamics could modulate the extent of material–tissue contact and the subsequent inflammatory response. This may partly explain why, in the absence of cryotherapy, lower pain scores were observed for BC Sealer compared with BC RRM toward the end of the postoperative period.

Recent evidence from a randomized clinical trial [36] indicates that not only cold saline but also cryotreated sodium hypochlorite can significantly reduce postoperative pain, with significantly lower pain scores, particularly during the early postoperative period. This finding suggests that the temperature of the irrigant itself may play a critical role in modulating periapical inflammation and nociceptive response. While the present study applied cryotherapy as a final irrigation step using cold saline, the findings of this trial indicate that extending the cryotherapeutic effect throughout the irrigation phase may further enhance early pain control. However, unlike the present study, the analgesic effect observed in cryotreated NaOCl studies appears to be more pronounced in the immediate postoperative period. This difference may be attributed to variations in cryotherapy protocols, irrigant composition, and duration of application. Taken together, these findings suggest that intracanal cryotherapy may exert both immediate and sustained analgesic effects depending on how and when it is applied during the treatment protocol. While cryotreated NaOCl may provide an early-phase benefit, the present findings indicate that final irrigation with cold saline may contribute to a more prolonged modulation of postoperative pain. A key strength of this study is the high level of procedural standardization achieved by including only cases treated according to a uniform clinical protocol, ensuring consistency in instrumentation size, irrigation regimen, obturation procedures, and postoperative pain assessment at predefined time points. This methodological consistency minimized procedural variability and enhanced the internal validity of the findings, allowing a more reliable evaluation of the effects of intracanal cryotherapy and obturation materials on postoperative pain. However, given the retrospective design and reliance on routinely recorded clinical data, the findings should be interpreted with caution. The absence of predefined randomization or stratification is an important methodological limitation, as both obturation material selection and the application of intracanal cryotherapy were based on routine clinical decision-making and operator preference. Therefore, selection bias and unmeasured confounding cannot be excluded, particularly if treatment choices were influenced by clinical factors not captured in the retrospective records. For this reason, the observed differences should be interpreted as associations rather than evidence of causal effects. Furthermore, although the study population was restricted to single-rooted, single-canal teeth diagnosed with irreversible pulpitis to enhance sample homogeneity, the inclusion of different tooth types may still have limited complete standardization of the experimental conditions due to anatomical and biomechanical variations. An additional limitation of the present study is the potential floor effect observed in VAS pain scores at 48 and 72 h postoperatively, where several groups demonstrated median values of 0 (0). Although these findings may reflect substantial postoperative recovery and minimal residual pain, clustering at the lower end of the scale may have reduced the sensitivity of the VAS instrument to detect subtle differences in discomfort between groups during the late postoperative period. Future studies incorporating complementary pain assessment tools or more granular outcome measures may provide greater discriminatory capacity in patients with very low pain levels. Future randomized clinical trials employing a split-mouth design, larger sample sizes, and a single tooth type are recommended to further clarify the effects of cryotherapy and obturation-related variables on postoperative pain.

## 5. Conclusions

Intracanal cryotherapy may serve as an adjunctive approach associated with lower postoperative pain scores across obturation materials with differing chemical compositions and physical properties. These findings suggest that cryotherapy may represent a practical, low-cost strategy to enhance early patient comfort in routine endodontic care. Under cryotherapy conditions, the bioceramic-based groups showed lower pain scores than the epoxy resin-based group, indicating a possible material-related trend in later postoperative pain outcomes following single-visit root canal treatment.

## Figures and Tables

**Table 1:** Comparison of postoperative pain between cryotherapy and non-cryotherapy groups.

	Cryotherapy (−) (*n* = 35)	Cryotherapy (+) (*n* = 38)	
	Median (IQR)		*p*
Day 1	40 (18) ^A,b^	20 (21) ^B,b^	<0.001 ^1^
Day 2	20 (10) ^A,c^	0 (0) ^B,c^	<0.001 ^1^
Day 3	10 (20) ^A,d^	0 (0) ^B,c^	<0.001 ^1^
*p*	<0.001 ^2^	<0.001 ^2^	

IQR: Interquartile range. ^1^ Mann–Whitney U test. ^2^ Friedman test with Durbin–Conover post hoc test. Different uppercase and lowercase letters denote significant differences within rows and columns, respectively (*p* < 0.05).

**Table 2:** Combined effects of obturation material and intracanal cryotherapy on postoperative pain.

	AH Plus		TotalFill BC RRM		TotalFill BC Sealer		
	Cryotherapy (−) (*n* = 11)	Cryotherapy (+) (*n* = 11)	Cryotherapy (−) (*n* = 11)	Cryotherapy (+) (*n* = 13)	Cryotherapy (−) (*n* = 13)	Cryotherapy (+) (*n* = 14)	*p*
	Median (IQR)						
Day 1	45 (35) ^A,b^	20 (3) ^B,b^	40 (18) ^A,b^	20 (30) ^B,b^	30 (20) ^A,b^	20 (24) ^B,b^	<0.001 ^1^
Day 2	20 (25) ^A,c^	10 (20) ^A,c^	20 (10) ^A,c^	0 (0) ^B,c^	20 (0) ^A,c^	0 (0) ^B,c^	<0.001 ^1^
Day 3	20 (10) ^A,c^	0 (0) ^B,d^	10 (20) ^A,d^	0 (0) ^B,c^	0 (10) ^B,d^	0 (0) ^B,c^	<0.001 ^1^
*p*	<0.001 ^2^		<0.001 ^2^		<0.001 ^2^		

IQR: Interquartile range. ^1^ Kruskal–Wallis test with Dwass–Steel–Critchlow–Fligner post hoc test. ^2^ Friedman test with Durbin–Conover post hoc test. Different uppercase and lowercase letters denote significant differences within rows and columns, respectively (*p* < 0.05).

## Data Availability

Data is contained within the article or Supplementary Materials.

## Supplementary Materials

The following supporting information can be downloaded at: https://www.mdpi.com/article/10.3390/jcm15103899/s1, Supplementary Table S1: Patient follow-up form.

## Abbreviations

The following abbreviations are used in this manuscript:

BC	Bioceramic
PROBE	Preferred Reporting items for Observational studies in Endodontics
RRM	Root repair material
VAS	Visual Analog Scale
