# Supplementary material for: Cryotherapy-Driven Modulation of Postoperative Pain in Single-Visit Endodontic Treatment Across Different Obturation Materials: A Retrospective Study
Source: J Clin Med. 2026 May 19;15(10):3899. doi: 10.3390/jcm15103899 (PMC13206843; doi:10.3390/jcm15103899)
Supplement: Supplementary file 1 [file jcm-15-03899-s001.zip › jcm-4287201-supplementary.pdf]

Table S1: Patient follow-up form

Date	
Name	
Phone	
Age	
Gender	F/M
Medical History	
Dental History	
Diagnosis	
Swelling	
Sinus Tract	
Tooth	
Vitality	
Periapical Status	
Percussion	/ 10
Palpation	+ / -
Irrigation Solutions	NaOCl / EDTA / CHX / SF
Routine Irrigation Protocol	+ / -
Cryotherapy	+ / -
Irrigation Activation	
Obturation Material Type	
Intracanal Medicament	
Type of Treatment (please specify additional procedures performed; e.g., resorption repair, regeneration, etc.)	
File System Used / Final File	
Presence of Pre-operative Complications	
Did a Complication Occur During the Procedure?	
Session	
Restoration	
Preoperative VAS Score	
Postoperative VAS Scores at 8, 16, 24, 48, and 72 Hours	/ / / /
Analgesic/Antibiotic Use	Pre-op: + / - Post-op: + / -
Type and Count of Anesthesia	Regional/ Infiltrative
Procedure Duration	
Operator Performing the Procedure	
